# Pathophysiological Implications of Different Bicuspid Aortic Valve Configurations

**DOI:** 10.1155/2012/735829

**Published:** 2012-05-27

**Authors:** F. A. Kari, F. Beyersdorf, M. Siepe

**Affiliations:** Department of Cardiovascular Surgery, Heart Center Freiburg University, Hugstetter Stra*β*e 55, 79106 Freiburg, Germany

## Abstract

There are numerous types of bicuspid aortic valve (BAV) configurations. Recent findings suggest that various BAV types represent different pathophysiological substrates on the aortic media level. Data imply that the BAV type is probably not related to location and extent of the aneurysm. However, BAV type is likely linked to the severity of aortic media disease. Some BAVs with raphe seem more aggressive than BAV without a raphe. Cusp fusion pattern, altered hemodynamics, and the qualitative severity of the disease in the aortic media might on the one hand share the same substrate. On the other hand, the aortopathy's longitudinal extent and location may represent a different pathophysiological substrate, probably dictated by the heritable aspects of BAV disease. The exact nature of the relation between BAV type and the aneurysm's location and extent as well as to the risk of aortic complications remains unclear. This paper reviews results of recent human and experimental studies on the significance of BAV types for local aortic media disease and location and extent of the aortopathy. We describe the known and hypothesized hemodynamic and hereditary factors that may result in aortic aneurysm formation in BAV patients.

## 1. Introduction

A substantial number of individuals with bicuspid aortic valve (BAV, 1-2% of the population) never develop any symptoms or complications. However, the bicuspid aortic valve (BAV) is accompanied by an intrinsic structural defect of the aortic media which leads to fragmentation and rarefaction of elastic fibers and subsequent aortic dilation in 40–60% of patients suffering from symptomatic or complicated BAV disease. This process involves the aortic root, ascending aorta, and, in up to 70% of BAV patients, portions of the aortic arch [1–3]. It is associated with different cardio-vascular malformations like aortic coarctation, ventricular septal defect, patent ductus arteriosus, or Shone's complex [2, 4, 5]. In addition, BAV is associated with hereditary syndromes like Turner's. However, the most frequently observed associated pathologies are aneurysms of the aortic root, ascending aorta, and aortic arch, as well as aortic complications like acute dissection [6–9].

The nature of the pathophysiological interrelations between BAV configuration and extent and severity of the associated aortic aneurysm, as well as the risk of possible complications, is of great immediate interest to the clinician. It is often unclear which patient can be treated by valve surgery only, especially when reconstruction is a feasible option. Aortic size criteria prompting additional ascending aortic replacement and root replacement or reconstruction are subject to ongoing discussion. Understanding the details of the complex interplay between BAV configuration and associated aortopathy would form the basis for methods to identify patients at risk for aortic dilation and subsequent aortic complications.

As is the case with many associated malformations, there is a wide variety of pathological configuration patterns of the cusps, sinuses, and commissures in bicuspid aortic valve disease. This has been systematically approached by categorizing BAV according to the raphes' (fusion of cusps, “seam” or “rim”) number and location with respect to the three cusps, also including valve function. Sievers and Schmidtke introduced three main categories and 24 subcategories of potential BAV configuration types. The different kinds of bicuspidization of the aortic valve are thought to represent a continuous pathological spectrum from the normal tricuspid valve to the “naturally perfect” BAV (termed by the Stanford-group) with two cusps, two commissures, and two sinuses, to valves with one raphe (and three retained sinuses), and, ultimately, valves with two raphes and a unicuspid aortic valve (UAV) [10]. However, it is unclear whether the true unicuspid valve should actually be described as a BAV with two raphes, as is the case in Sievers' classification system, or whether the UAV from a developmental point of view represents a separate pathology. Little is known about the embryological process of nonseparation of cusps in BAV disease and even less for UAV disease. Sievers' type 0 BAV, with the number representing the number of raphes, is the BAV with two cusps, two commissures, and two sinuses sometimes called “naturally perfect.” This type of valve is found with both sinuses in an anterior-posterior (“ap”) direction or in a lateral orientation (“lat”). The type 1 BAV can be observed with one raphe between right and left coronary cusps (R-L, behind the posterior commissure of the pulmonic valve), and also between the right and non- (R-N, in close proximity to the septal commissure of the tricuspid valve) and between the non- and left coronary sinuses (N-L, right above the medial scallop of the anterior mitral valve leaflet). Certain configurations are more common than others and are thus considered “majority type” as is type 1 BAV with fusion of the right and left coronary cusps, presenting with one raphe (Sievers 1 right-left, referred to as S1/R-L type). Others are considered “minority types” as is the “naturally perfect” BAV without a raphe (S0), or the valve presenting with one raphe and fused right and non-coronary cusps (S1/R-N).

In addition to the Sievers' type, there are other features of the bicuspid aortic valve which may affect the aortic root and ascending aortic hemodynamics. First, the angle of circumferential orientation of the free (nonfused) commissures differs substantially between valves, normally ranging somewhere between 140 and 180 degrees. Secondly, the completeness of cusp fusion of BAV with raphe (complete versus incomplete raphe) might have an impact on hemodynamics and be interrelated with the development, location, and extent of the aortic aneurysm [11].

It seems reasonable to hypothesize that differently configured BAV, for example, an S0 BAV versus an S1/R-L BAV with completely fused cusps, could affect blood flow patterns in the aortic root and ascending aorta differently. Altered hemodynamics might lead to different wall strain, shear, and other stress factors and affect the aortic media's integrity differently. They might be the reason behind aortic aneurysm development and a major factor in how the aortic aneurysm's expansion progresses. On the contrary, genetic factors may likewise cause different types of BAV formation and changes in the media, so that the hemodynamics may be secondary (Tables 1 and 2). In this paper we highlight the implications of various BAV types regarding the nature of the associated aortic aneurysm.

## 2. Pathophysiological Consequences of Different Bicuspid Aortic Valve Configurations

Several recent reports reveal that the different BAV configuration types are both patho-morphologically distinct and that they may reflect distinctly different disease processes with respect to molecular aortic media pathology. Ikonomidis et al. hypothesized that each BAV configuration type has a unique “signature” of local Matrix-Metalloproteinases (MMPs) and Tissue Inhibitors of Matrix-Metalloproteinases (TIMPs) expression patterns. Using a categorization system similar to Sievers' classification in a human aortic tissue study, they found (in addition to elevated global MMP activity in all BAV types compared to a tricuspid valve (TAV) aortic specimen) that different BAV configuration types did indeed exhibit different expression patterns of MMPs (types 7, 8, and 9) and TIMPs (types 1 and 4) [12].

The causative relations, however, between BAV configuration type and differently expressed local protein patterns remain unclear. Investigators reporting a different human study, shifting the focus from local MMP expression to circulating levels of MMPs and their tissue inhibitors, succeeded in showing that the presence of a BAV correlates quantitatively with the circulating amount of those proteins. Den Reijer et al. demonstrated that acute-outflow jet angles from the left ventricular outflow tract correlate with increased circulating levels of MMP-2 and MMP-9 and their TIMPs, as well as with more severe aortic root and ascending aortic dilation [13]. It has, however, not been investigated whether certain BAV types actually correlate with different circulating MMP and TIMP measurements, using a detailed categorization system for BAV configurations. Furthermore, we do not know if increased local aortic media protein expression is in fact related in any way to circulating plasma levels. Moreover, it remains to be studied whether local MMP and TIMP expression will actually translate into clinically significant differences in aortic complication rates. Locally increased MMP and TIMP expression can probably be interpreted as markers of the local immune response, cell turnover, matrix degradation, and greater overall disease activity. Understanding those key factors will form the basis for evaluating circulating MMP and TIMP levels and their potential importance as aortic biomarkers.

We can hypothesize that bicuspid valve morphology is tightly bound to functional aortic root parameters such as jet direction, flow acceleration and velocity, and the resulting mechanical forces on the aortic root and ascending aortic wall. It would therefore be reasonable to assume that different BAV types could affect qualitative aortic wall damage differently [14]. Interestingly, recent data suggest that the respective BAV type does play a role in the degree of local aortic wall pathology.

The “majority type” BAV with one raphe and fused left and right coronary cusps seems to affect flow mechanics in a particularly devastating manner (two of the sinuses are often normally sized, one often tends to be degenerated). Using a histopathological grading score in human tissue samples, this type of BAV has been shown to correlate with a more severe degree of ascending aortic wall degeneration compared to “minority type” BAV types like the Sievers' types 1/R-N and 1/N-L valves [14]. Furthermore, aneurysm development occurred at younger age in the “majority type” BAV cohort in this human tissue sample study of 115 individuals. One can only speculate why there is such an interrelation, as there is no evidence proving a causative association. It is, however, likely that the presence of a raphe leads to decreased fused cusp mobility. We can thus assume there are more excentric aortic root flow jets. In turn, excentric and accelerated flow in the aortic root might lead to higher mechanical burden on the aortic root and ascending aortic intima. In fact, reduced fused cusp mobility has been reported to be an independent predictor of aortic aneurysm development, thus adding support to this hypothesis.

Moving a step further from aortic valve morphology to valve function, a human tissue sample study was carried out by Roberts et al. investigating ascending aortic media elastic fiber loss and comparing different groups of valve function. According to their results, valve function (aortic stenosis versus aortic regurgitation) correlates with the severity of qualitative media aortopathy and elastic media fiber loss [15]. Loss of elastic fibers may be directly linked to higher rates of aortic complications in patients with BAV, but this remains to be proven in further studies. Aortic root dilation is most common in BAV patients with a predominantly regurgitant valvular lesion [16], but we do not yet know why this is so.

Several attempts have been undertaken to study not just local histopathological damage and quantity of aortic media disease, but also the location and extent of the aortic aneurysm in BAV disease. We know that the aortic arch is involved in about 70% of BAV patients [1]. On the other hand, there is a large proportion of patients with aortic root aneurysm only or without aneurysms of the thoracic aorta and BAV. The question whether certain BAV types are interrelated with the development of particular “clusters” of aortopathy, as described by Fazel et al., is of scientific interest and considerable clinical significance. So far, sparse data indicate that bicuspid aortic valve morphology probably does not predict the pathologic anatomy of the thoracic aorta. In their study with a large cohort (*n* = 300), Jackson et al. analyzed echocardiographic BAV evaluation in conjunction with intraoperative evaluation of valve morphology, identifying aorta dilation patterns distributed similarly regardless of BAV type [17]. Moreover, Fazel et al. identified no such interrelations in their original investigation of BAV-associated aortopathy defining “clusters” of BAV-associated aneurysms [1].

It should be mentioned that BAV types and morphological valve details beyond the criteria of the classification introduced by Sievers et al. are also potential predictors of medium- and long-term results after surgery for BAV disease. A recent report by Schäfers et al. claims that BAV configuration types influence results after reconstructive surgery of the aortic root and aortic valve. For example, an incomplete raphe seems to be associated with worse medium-term outcome after BAV reconstruction [11, 18]. Furthermore, BAVs of small circumferential free commissural orientation angle (<160°) might be associated with worse medium-term functional outcome after bicuspid aortic valve repair [11].

## 3. Intrinsic Structural Media Defect in Bicuspid Aortic Valve Disease

Major scientific efforts have recently been made to clarify the nature and pathomechanisms of the intrinsic structural media defect in bicuspid aortic valve disease (the nature of the media defect has been described by others [4]). For example, the GenTAC Registry, with 25% BAV patients, included validation studies of genetic causes for different hereditary aortic syndromes and the usefulness of potential biomarkers like plasma levels of transforming growth factor beta (TGFB) [19]. Although the discussion of pathological mechanisms in BAV disease and aneurysm development is often described as a two-sided debate [9], evidence suggests that hemodynamic and structural abnormalities both exist and go hand in hand in complex BAV pathophysiology. Interesting data of late implies hereditary intrinsic aortic media disease in BAV patients. As pathological cusp configuration has been shown to be closely linked to the severity of local media disease, an additional hereditary component of BAV disease might predetermine the extent and location of the associated aortic aneurysm, including arch involvement.

Biner et al. conducted a study on elastic properties of the aortic roots in first-degree relatives of patients with a BAV (*n* = 54) and found that even in those BAV patients' relatives who presented a tricuspid aortic valve, their aortas were stiffer, less compliant, and somewhat enlarged [20]. In their report on a low-volume follow-up study, Yasuda et al. describe that after isolated BAV replacement, aortic dilation progresses and the risk of aortic rupture and dissection can remain higher [21]. In light of an optimal hemodynamic profile on the valvular level after replacement, this finding needs further clarification. The presence of a BAV independently influences the proximal aortic diameter, as shown by Martin et al. in a recent report on their large heritability study involving the assessment of 209 families [22]. Using variance components analysis, heritability was estimated with and without BAV status. Aortic diameter *per se* is a quantitative trait that exhibits significant familial heritability—moreover, they found the bicuspid aortic valve to be an independent modifier.

Genetic “fingerprints” of the aortic media in BAV disease have been the focus in several recently published studies. Alternative splicing of certain genes is common in thoracic aortic aneurysms [23]. Those genes involve coding sequences for structural components of the extracellular vascular matrix (ECM), as well as an important system in ECM repair, the TGF-beta pathway. Recently reported results describe the identification of diverging alternative splicing of the TGF-beta signaling pathways in BAV patients. Differential splicing is specific for BAV and TAV patients in 40 and 86 exons when an aneurysm is present. 61 exons were found to be shared between the two valvular phenotypes by Kurtovic et al. [23]. Their group proved the occurrence of differential splicing in selected genes by reverse transcription-polymerase chain reaction. Aortic aneurysms in TAV and BAV patients have different alternative splicing fingerprints in the TGF-beta pathway. The pathways of TGF-beta and downstream Smad2 signaling have been found to be subject to epigenetic control in thoracic aortic aneurysm patients (about a third had BAV in the study by Gomez et al. [24]).

Not only the tissue derived growth factor protein itself, but also molecular coworkers in the complex signaling pathways of this seemingly crucial molecule seem to be of significance in BAV disease. Endoglin is a membrane glycoprotein on many cell surfaces and has been identified on endothelial cells. It is a functional part of the TGF-beta1 receptor complex and thus is thought to play a major role in TGF-beta signaling. A specific haplotype of this glycoprotein has recently been found to be strongly linked to BAV using gene network analysis techniques [25]. In addition, Paloschi et al. reported defective fibronectin splicing within the aortic wall of bicuspid aortic valve to be associated with aortic aneurysm development [26]. Other genetic polymorphisms related to aortic aneurysm development include the ACE insertion/deletion polymorphism—another possible genetic biomarker for thoracic aortic aneurysm [27]. The evolving field of biomarker research in aortic disease might become of great clinical significance in the future [28, 29].

The comparative analysis of gene expression profiles of aneurysms in TAV and BAV patients by Folkersen et al. provides initial evidence of fundamental differences in aortic aneurysm etiology in BAV and TAV patients [30]. They observed that immune response genes are particularly overexpressed in the aortic media of dilated TAV aortic human samples. The fact that immune response activation was solely found in the aortic media of TAV patients suggests that inflammation is involved in aortic aneurysm formation in TAV but not BAV patients. There is still no reliable diagnostic factor to clarify whether a patient with BAV has the disadvantage of inferior wall configuration with the risk of aneurysm formation. Intense research is focusing on identifying this clinically relevant prognostic parameter.

## 4. Conclusions

The numerous different configuration types of the bicuspid aortic valve (BAV) are likely to be interrelated with the histopathological severity of aortic media disease. Recently published results suggest, however, that the BAV type is probably not directly related to the location and extent of the aortic aneurysm, including arch involvement. The relation of BAV types to occurrence, location, and extent of the aortic aneurysm and to the risk of aortic complications requires further intense investigation. The majority type (Sievers') 1/L-R valve seems to be a more aggressive BAV type when compared to other Type 1 and Type 0 bicuspid aortic valves and is probably linked to more severe hemodynamic alterations and aortic media disease. There might be different pathophysiological substrates for cusp fusion patterns, altered hemodynamics, and the qualitative severity of disease in the aortic media on the one hand. On the other hand, the aortopathy's longitudinal extent, location, and arch involvement may represent a different pathophysiological substrate, probably dictated by the heritable aspects of BAV disease. As we hope to be able to advise our patients undergoing surgery on the BAV much better in the future, we should base our decision to replace the aorta not only on its size or appearance during surgery, but on other proven risk factors for future pathology and risk of complications as well.

## Figures and Tables

**Table 1:** Cusp fusion pattern—local hemodynamics—qualitative severity of aortic media disease.

Different local aortic MMP and TIMP patterns in different types of BAV [12]	
Presence of BAV correlates with circulating MMP and TIMP levels [13]	
Majority type S1-RL BAV linked to more severe media disease [14]	

**Table 2:** Hereditary factors—longitudinal extent—arch involvement of aortopathy.

Aortic dilation patterns are distributed similarly regardless of BAV type [1, 17]	
BAV patients' relatives with tricuspid aortic valve have stiffer, less compliant, and somewhat enlarged aortas [20]	
After isolated BAV replacement, aortic dilation progresses and the risk of aortic rupture and dissection remain higher [21]	
Aortic diameter *per se* is a quantitative trait that exhibits significant familial heritability—bicuspid aortic valve is independent modifier [22]	
TGF-beta signaling—differential splicing is specific for BAV and TAV patients [23]	
Defective fibronectin splicing within the aortic wall of bicuspid aortic valve is associated with aortic aneurysm development [26]	
ACE insertion/deletion polymorphism associated with aneurysm formation [27]	
